# A novel barcoded nanopore sequencing workflow of high-quality, full-length bacterial 16S amplicons for taxonomic annotation of bacterial isolates and complex microbial communities

**DOI:** 10.1128/msystems.00859-24

**Published:** 2024-09-10

**Authors:** Julian Dommann, Jakob Kerbl-Knapp, Diana Albertos Torres, Adrian Egli, Jennifer Keiser, Pierre H. H. Schneeberger

**Affiliations:** 1 Department of Medical Parasitology and Infection Biology, Swiss Tropical and Public Health Institute, Allschwil, Switzerland; 2 University of Basel, Basel, Switzerland; 3 Institute of Medical Microbiology, University of Zurich, Zurich, Switzerland; 4 Clinical Bacteriology and Mycology, University Hospital Basel, Basel, Switzerland; 5 Applied Microbiology Research, Department of Biomedicine, University of Basel, Basel, Switzerland; Iowa State University, Ames, Iowa, USA

**Keywords:** DNA sequencing, gut microbiome, bioinformatics

## Abstract

Due to recent improvements, Nanopore sequencing has become a promising method for experiments relying on amplicon sequencing. We describe a flexible workflow to generate and annotate high-quality, full-length 16S rDNA amplicons. We evaluated it for two applications, namely, (i) identification of bacterial isolates and (ii) species-level profiling of microbial communities. We assessed the identification of single bacterial isolates by sequencing, using a set of barcoded full-length 16S rRNA gene primer pairs (pair A), on 47 isolates encompassing multiple genera and compared those results with matrix-assisted laser desorption ionization-time of flight mass spectrometry (MALDI-TOF MS)-based identification. Species-level community profiling was tested with two sets of barcoded full-length 16S primer pairs (A and B) and compared to the results obtained with shotgun Illumina sequencing using 27 stool samples. We developed a Nextflow pipeline to retain high-quality reads and taxonomically annotate them. We found high agreement between our workflow and MALDI-TOF data for isolate identification (positive predictive value = 0.90, Cramér’s *V* = 0.857, and Theil’s *U* = 0.316). For species-level community profiling, we found strong correlations (*r*_*s*_ > 0.6) of alpha diversity indices between the two primer sets and Illumina sequencing. At the community level, we found significant but small differences when comparing sequencing techniques. Finally, we found a moderate to strong correlation when comparing the relative abundances of individual species (average *r*_*s*_ = 0.6 and 0.533 for primers A and B). Despite identified shortcomings, the proposed workflow enabled accurate identification of single bacterial isolates and prominent features in microbial communities, making it a worthwhile alternative to MALDI-TOF MS and Illumina sequencing.

**IMPORTANCE:**

A quick, robust, simple, and cost-effective method to identify bacterial isolates and communities in each sample is indispensable in the fields of microbiology and infection biology. Recent technological advances in Oxford Nanopore Technologies sequencing make this technique an attractive option considering the adaptability, portability, and cost-effectiveness of the platform, even with small sequencing batches. Here, we validated a flexible workflow to identify bacterial isolates and characterize bacterial communities using the Oxford Nanopore Technologies sequencing platform combined with the most recent v14 chemistry kits. For bacterial isolates, we compared our nanopore-based approach to matrix-assisted laser desorption ionization-time of flight mass spectrometry-based identification. For species-level profiling of complex bacterial communities, we compared our nanopore-based approach to Illumina shotgun sequencing. For reproducibility purposes, we wrapped the code used to process the sequencing data into a ready-to-use and self-contained Nextflow pipeline.

## INTRODUCTION

Identifying bacteria and resolving the relationship between microbial communities and their environment has shed light on the importance of bacterial communities in both health and disease (1–3). The potential to utilize microbes as research subjects to comprehend, characterize, and treat diseases helped drive innovative techniques such as DNA sequencing (4, 5). Currently, among the different methods to sequence DNA, ONT sequencing has emerged as a novel approach for the high-throughput identification of bacteria (6, 7). Although initial drawbacks in accuracy (few correctly aligned bases between generated sequences and reference genomes) limited the robustness and applicability of this method, continuous advances in kit chemistry, flow cell design, basecaller updates, and available bioinformatics tools have drastically increased the usability of the ONT sequencing platform (8–11). Improvements in basecalling yield high-quality reads, whereas automatable data processing allows simultaneous processing of large numbers of samples. With the recent v14 chemistry kits, a Phred score of 20 (accuracy of 99%) is achievable in 70%–80% of the reads (12), turning ONT sequencing into a robust, affordable, and particularly versatile option for bacterial identification, suitable even for field work (13–15). For instance, Urban et al. (16) recently demonstrated the utility of the ONT platform for the investigation of large numbers of bacterial samples, employing custom barcoded 16S PCR primers to increase the throughput of the platform while containing costs. Building upon this approach, it is desirable to develop an end-to-end 16S amplicon sequencing workflow that is (i) easily implementable, (ii) adaptable and scalable to diverse microbiological samples at contained costs, and (iii) utilizes the latest v14 kit chemistry and Flongle Flow Cells (R10.4.1).

Here, we first introduce and validate a step-by-step workflow for either a single or double barcoding approach using ONT sequencing. Our workflow relies on a full-length, barcoded 16S amplicon PCR prior to ONT library preparation. Consequently, a pair of either identical (1BC) or different (2BC) barcodes were attached to a given 16S amplicon, flanking the primer binding sites. Barcoded amplicons were then pooled to serve as one library preparation input sample. Second, we developed a flexible data processing pipeline, involving either simplex (S1BC or S2BC) or duplex (D1BC or D2BC) basecalled data for higher sequencing depth or sequencing accuracy, respectively. The code is available as a Nextflow pipeline based on validated bioinformatics tools enabling filtering, trimming, demultiplexing, and taxonomic annotation of the sequenced 16S rDNA amplicons.

## MATERIALS AND METHODS

### Sample selection

We initially sequenced 47 bacterial isolates, for which identification results via matrix-assisted laser desorption ionization-time of flight mass spectrometry (MALDI-TOF MS) were available. We sequenced another 97 bacterial isolates to further explore the potential of our sequencing approach regarding phylogenetic resolution. In total, we sequenced 144 bacterial isolates, spanning across 41 species and 20 genera. While not all isolates are of medical relevance, we enhanced taxonomic diversity by including both phylogenetically distant and close species, both aerobic and anaerobic species, and isolates originating from different sample types (for instance from blood or stool). The 144 bacterial isolates tested in this study are listed in Table S1. Bacterial stocks in 20% glycerol were stored at −80°C. To assess the performance of species-level microbiome characterization, we sequenced DNA extracts of 27 human stool samples gathered from individuals recruited as part of a multi-country randomized controlled trial (NCT03527732) (17). For each sample, a small aliquot of stool (~1 g) was transferred to a sterile 2 mL cryotube and immediately frozen at −20°C. Upon completion of the trial, these aliquots were shipped to Swiss TPH (Allschwil, Switzerland) on dry ice and kept at −80°C until DNA extraction. Only fully anonymized samples and isolates were used throughout the study and no participant-specific information was available.

### Bacterial cultivation

Growth media were prepared using Milli-Q water and subsequently autoclaved at 121°C. Brain Heart Infusion broth and 5% yeast or modified Gifu anaerobic medium were used exclusively to cultivate all bacterial isolates. To cultivate an isolate, 10 µL of thawed glycerol stock was used to start a culture in 10 mL growth medium. To cultivate under anaerobic conditions, a vinyl anaerobic chamber (Coy Laboratory Products, MI, USA) with a gas mix composed of 85% N_2_, 10% CO_2_, and 5% H_2_ was used. Prior to working, the anaerobic chamber was cleaned using a 1:750 dilution of benzalkonium chloride (distribution from C_8_H_17_ to C_16_H_33_) in purified water to avoid cross-contamination. Aerobic isolates were handled in a safety cabinet (SKAN Berner, Elmshorn, Germany). All inoculated isolates were grown at 37°C. The growth of isolates was inspected daily, turbid cultures were pelleted at 3,000 rcf, the supernatant was removed up to 1 mL, and stored at −20°C.

### DNA extraction

Bacterial pellets were thawed at room temperature, and DNA was extracted using a commercially available extraction kit (DNeasy PowerSoil Pro Kit, Qiagen, Germany) according to the manufacturer’s protocol. Deviating from the protocol, 200 µL resuspended bacterial pellet was used as input, and the DNA was ultimately eluted in 60 µL C6 elution buffer to maximize yield while keeping the DNA concentration high. For the microbiome samples, ~100 mg of the stool sample was processed with the same kit using the same steps. DNA concentration and purity of all samples were measured using a Qubit 4 Fluorometer and the dsDNA BR Assay kit (both Invitrogen, USA)

### Barcoded 16S rDNA amplification

The underlying design of our 16S primer sequences was published by Urban et al. (16). Our adapted primers (primer pair A) contain a barcode sequence (see Table S2) that was derived from Illumina 12 bp barcode sequences published by Caporaso et al. (18). We also tested full-length 16S primers, recently published by Matsuo et al. (19), utilizing degenerate bases to resolve PCR bias and taxonomic underrepresentation of *Bifidobacterium* spp. (primer pair B) (Table S3). A and B primers were ordered from Microsynth (Balgach, Switzerland).

**Fig 1:**
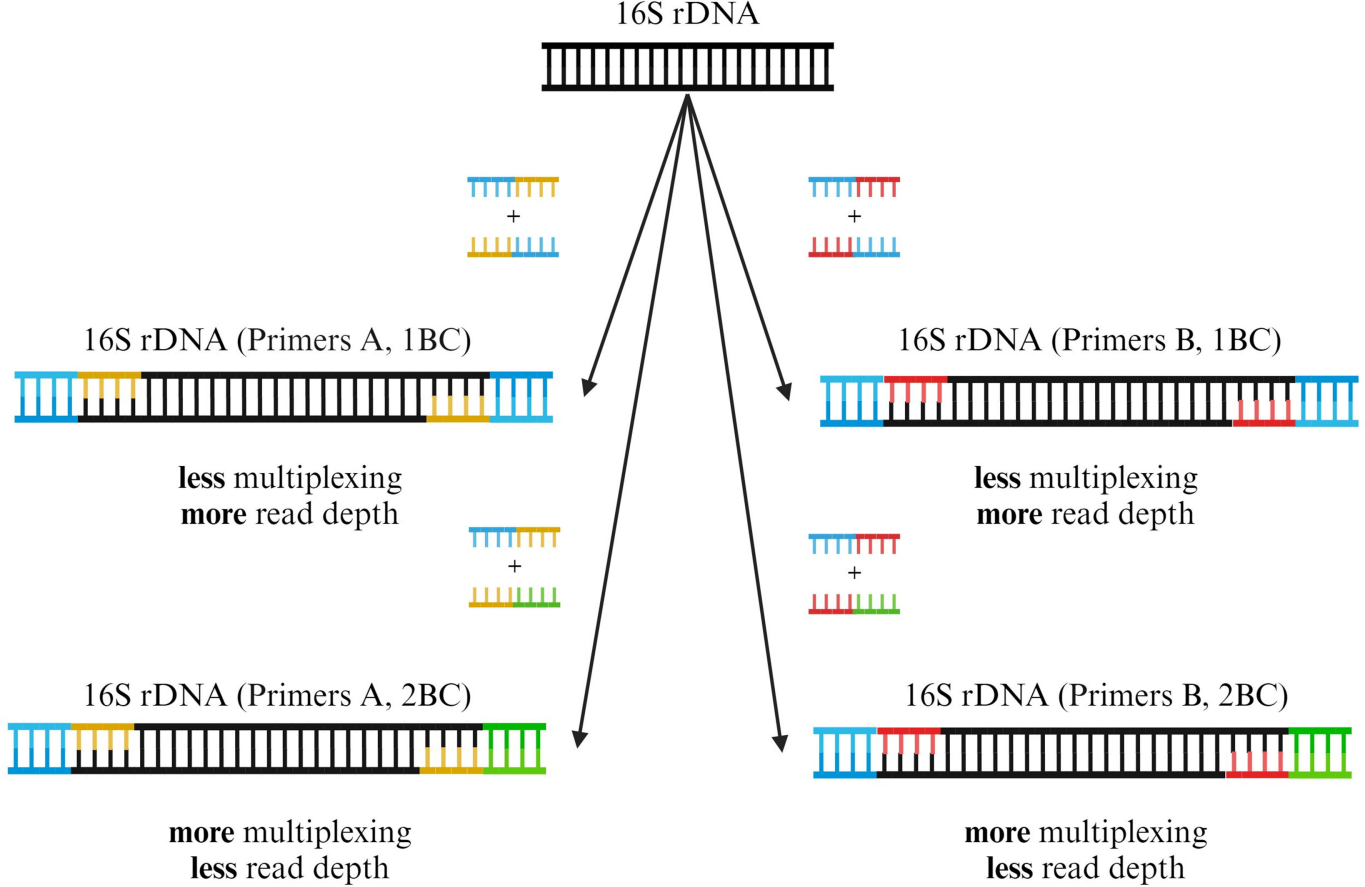
Barcoded 16S rDNA amplification using different primer pairs and barcodes (1BC and 2BC). Throughout this study, we tested two different barcoded primer pairs for 16S rDNA amplification (A or B). An exemplary 16S rDNA fragment is shown in black. Primer pair A (yellow) is derived from Urban et al. (16). Primer pair B (red) is derived from Matsuo et al. (19) and contains degenerate bases to enhance the amplification of *Bifidobacterium* spp. in complex microbiological samples. Forward and reverse primers for each primer set were constructed in eight different configurations, each consisting of the primer and one of eight unique barcode sequences. Depending on the desired multiplexing capabilities or sequencing depth, a 1BC (blue) or 2BC (blue and green) approach can be used during PCR preparation with either of the primer pairs A or B. Adding primers with two different barcodes (2BC) allows for a larger sequencing pool but will result in decreased read depth per sample. Utilizing the same barcode in the forward and reverse primers (1BC) results in decreased multiplexing capabilities but enhanced read depth per sample. Created with BioRender.com.

To acquire barcoded amplicons at high concentration, full-length 16S rDNAs of bacterial isolates or stool samples were amplified in a 96-well plate using one set of barcoded primer pairs (isolates: A; stool samples: A or B) (Fig. 1). The extracted DNA was first diluted 1:10 in nuclease-free water (Ultrapure Distilled water, Invitrogen, USA). A commercially available PCR Master Mix was used (LongAmp Hot Start Taq 2× Master Mix, New England BioLabs, USA). For each sample in a row, we utilized primer pairs with matching barcodes. For each plate column, forward and reverse primer pairs with different barcodes were used, yielding unique PCR barcode pairs for each sample in a plate column. Per-well reagents are presented in Table S4. The reaction was performed on an Eppendorf Mastercycler Nexus Gradient Thermal Cycler (Eppendorf, Germany) using a ramp rate of 1.5℃/s and cycling conditions as described in Table S5. DNA concentration of samples post-PCR was measured with the dsDNA BR Assay kit on a Qubit 4 Fluorometer (both Invitrogen, USA) according to the manufacturer’s protocol, using 2 µL of PCR reaction as input. Furthermore, the presence of PCR products and size were checked via gel electrophoresis in 1% agarose gels (100 V, 25 min). Sample plates were either stored at 4°C overnight or at −20°C for long-term storage.

### ONT library preparation and sequencing

Prior to ONT library preparation, barcoded amplicons were pooled column-wise from the 96-well plate in equimolar masses. Each pooled column consisting of eight individually barcoded amplicons served as one sample in the library preparation. Library preparation was done according to the protocol for the Native Barcoding Kit 24 v14 (SQK-NBD114.24, Oxford Nanopore Technologies, UK) to ensure a higher sample input of 200 ng, corresponding to 200 fmol of the expected 1.6 kb fragments, as opposed to the Native Barcoding Kit 96 v14 protocol. We applied the same library preparation protocol for both bacterial isolates and stool samples. Steps deviating from the protocol aimed at normalizing the sample input, purifying short fragments, and optimizing the final library for a Flongle Flow Cell (R10.4.1). They were as follows: (i) after the end repair, DNA concentration in the samples was measured using the 1× dsDNA HS Assay kit (Invitrogen, USA) on a Qubit 4 fluorometer. Equimolar masses of the samples were used as input for the native barcode ligation, which should lead to equal read numbers per native and PCR barcode. Dilutions were made with nuclease-free water. (ii) The adapter ligation and clean-up were done according to protocol; the short-fragment buffer was used to preserve the 16S rDNA fragments. (iii) The protocol was modified for usage with a Flongle Flow Cell (R10.4.1) as follows: for the priming of the Flongle Flow Cell, 3 µL Flow Cell Tether was added to 117 µL Flow Cell Flush. This mixture was used to prime the Flongle Flow Cell. The DNA concentration of the final library was measured using the Qubit 4 fluorometer (1× dsDNA HS Assay kit) and diluted to a concentration of 2–3 ng/µL using nuclease-free water. For the loading of the library, 15 µL sequencing buffer, 10 µL loading beads, and 5 µL of the final library were mixed and loaded onto a Flongle Flow Cell on a MinION Mk1C (Oxford Nanopore Technologies, UK). An overview of the sequencing run settings is described in Table S6. For the microbiome samples, each library was sequenced in triplicate on independent flow cells to mitigate sequencing bias and depth variations arising from flow-cell-specific differences in total sequencing output.

### Data processing and taxonomic annotation of Nanopore data

**Fig 2:**
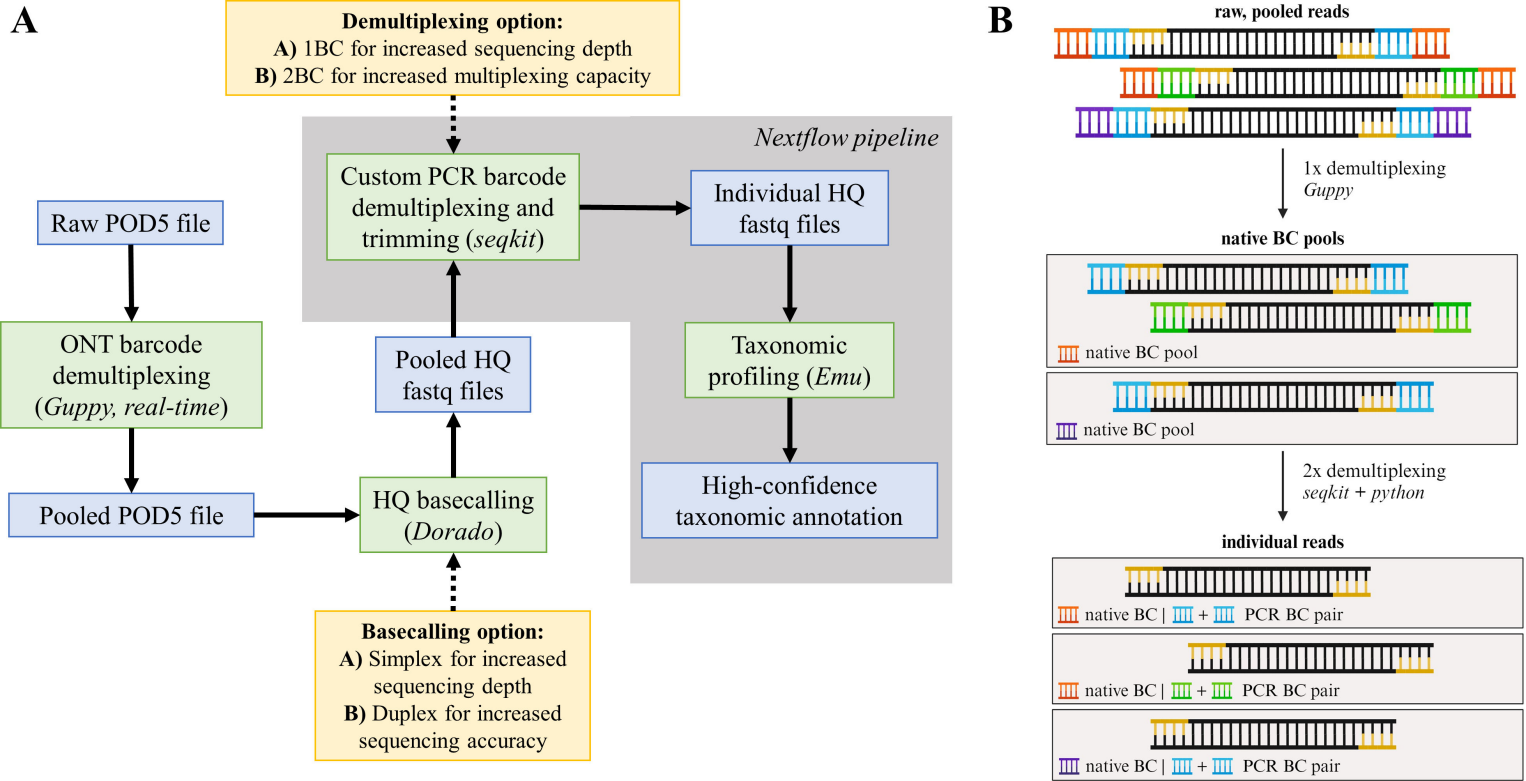
Schematic workflow of the used analysis pipeline. (A) POD5 files were demultiplexed in real-time using Guppy and basecalled via Dorado (simplex or duplex). Contained in the nextflow pipeline (gray), a combination of seqkit and custom Python scripts demultiplex the pooled fastq files based on the PCR barcodes to yield individual high-quality (HQ) fastq files. High confidence-taxonomic annotation was performed with the Expectation-Maximization algorithm implemented in the Emu software (23). Obtained files are marked in blue, processing steps in green, and processing options in yellow. (B) Overview of the demultiplexing strategy (BC = barcode). First, the raw, pooled reads are demultiplexed based on their native barcode (orange, purple) that was attached during ONT library preparation. The resulting native read pools are later once more demultiplexed via seqkit and custom Python scripts by their PCR barcode combination (blue, green) to yield individual reads. Primer sequences are depicted in yellow. Created with BioRender.com.

Read processing for bacterial isolate sequencing was done as follows (Fig. 2): the POD5 files generated for each sequencing run were demultiplexed based on the native ONT barcode, which were added during ONT library preparation, in real time utilizing the MinION’s built-in Guppy (version 6.5.7). The 1× demultiplexed POD5 files—still representing pooled amplicons with individual PCR barcodes (Fig. 2B; “native BC pools”)—were basecalled using Dorado (version 0.2.4; basecaller model “dna_r10.4.1_e8.2_400bps_hac@v4.1.0”). We performed read quality control via seqkit (version 2.6.1) (20). Basecalled reads served as input for a custom Nextflow (21) pipeline performing the following steps: first, reads are filtered based on quality and length using NanoFilt (22) (version 2.8.0; 1,300–1,800 bp, quality score > 9) (23). Subsequently, a combination of seqkit (version 2.6.1) (20) and custom Python scripts demultiplex and trim the reads based on 1 PCR barcode (1BC) or a PCR barcode combination (2BC). If the reads are demultiplexed based on 1BC, seqkit extracts reads containing the forward primer with the barcode sequence and the reverse primer. If the reads are demultiplexed based on 2BC, seqkit extracts reads with the forward primer with the barcode and the reverse primer with the barcode. Thus, reads that were barcoded via 1BC can also be demultiplexed in 2BC mode to assess its performance. Finally, the 2× demultiplexed reads—representative of individual samples—were used as an input for Emu (23) (version 3.4.5) and the default 16S rRNA database (based on rrnDB version 5.6 and NCBI 16S RefSeq from September 2020), for high-confidence species-level annotation. As an output, one receives the relative composition of each input sample depending on the similarity to the full-length 16S sequence. The Nextflow pipeline was built using singularity containers from https://depot.galaxyproject.org/singularity/. The methodology to process stool sample reads was identical to the processing of bacterial isolate reads except for the following differences: the demultiplexed POD5 files were basecalled using Dorado (version 0.5.0; basecaller model “dna_r10.4.1_e8.2_400bps_sup@v4.3.0”; simplex basecalling only) (24).

### Phylogenetic analysis

To generate per sample consensus sequences for the phylogenetic tree, we utilized vsearch (24) (version 2.23.0) and seqkit (20) (version 2.5.0) to generate read bins and centroids. Only bins yielding >10 reads were considered. Subsequently, ONT medaka (version 1.7.2) was used to generate consensus sequences for each bin and centroid. We used Blast+ (25) (version 2.13.0) to identify all consensus sequences and select the corresponding sequences for tree building in ClustalW2 (26) (version 2.1). Bootstrapping (*n* = 1,000) was performed in ClustalW2. The phylogenetic tree was visualized using MEGA-X (version 10.0.5) and iTOL v5 (27).

### Illumina sequencing and taxonomic profiling of microbial communities

For Illumina sequencing, isolated DNA from stool samples was processed as described in Schneeberger et al. (28). Briefly, samples were sequenced on a Novaseq platform to a depth of >5 M paired-end reads per sample in 2 × 150 bp PE mode. Kneadata (version 0.12.0) was used to filter out any remaining human reads. Metaphlan4 (version 4.0.4) (29), in combination with the CHOCOPhlAn database (vJan21_CHOCOPhlAnSGB_202103) and Bowtie2 (version 2.4.5) (30), was used to perform taxonomic profiling of the Illumina data sets. The resulting individual profiles were merged using the merge_metaphlan_tables.py script provided with the software.

### Cross-validation via MALDI-TOF MS

To prepare the samples for MALDI-TOF spectra measurement, Columbia agar plates with 5% sheep blood were streaked with the corresponding bacterial stocks. Colonies were left to grow in a CO_2_ incubator at 37°C, and single colonies were loaded onto a MALDI-TOF steel target plate using a sterile toothpick. Each colony on the plate was treated with 1 µL of 25% formic acid, left to dry, and then treated with 1 µL of α-Cyano-4-hydroxycinnamic matrix. After sample preparation, the plate was loaded into a microflex LRF MALDI-TOF mass spectrometer (Bruker, USA) for spectra measurement and analysis (MBT Compass reference library, version 2022). Only samples with ONT annotations > 90% purity (meaning 90% or more reads need to correspond to the same species in the Emu output) were considered and compared to MALDI-TOF MS results. To quantify the strength of the correlation between ONT and MALDI-TOF MS-based species annotation, we utilized R (version 3.4.1) and the R packages vcd (version 1.4), entropy (version 1.3.1), and ineq (version 0.2) to calculate Cramér’s *V* and Theil’s *U*. The positive predictive value was calculated by dividing the number of true positives (i.e., samples that were identified correctly via MALDI-TOF and our workflow) by the number of true positives plus false positives (i.e., samples that were differently identified by either workflow). As it was not possible to calculate the number of false negatives or true negatives and therefore sensitivity and specificity of our workflow, a PCR run of growth medium negative controls incubated and extracted under the same conditions as the bacterial inoculates was performed. Additionally, a heatmap using R (version 3.4.1) with the R package pheatmap (version 1.0.12) visualizing the average percent matches of the ONT reads for each species was generated.

### Processing of microbiome profiles

First, the processed reads of sequencing triplicates were pooled prior to annotation via Emu, resulting in one taxonomic profile for each sample. Second, due to the different databases used by the Emu and Metaphlan profilers, we matched the taxonomy of the 16S-based profiles (derived from both 16S primer sets) using the species identified by Illumina as the reference. Briefly, we used a set of criteria to systematically match non-matching species to create a unified abundance file containing all samples and conduct downstream analyses (e.g., analyses of beta diversity). The criteria and their corresponding results are summarized in Fig. S1. Alpha diversity, including the number of taxa, the Shannon diversity, and the Berger-Parker dominance index, was calculated for each sample using PAST (version 4.13) (31) and correlated using Spearman correlation available in the XL-STAT software (version 2023.2.1414) (Lumivero 2024, https://www.xlstat.com/en). The Bray-Curtis index was used to measure beta diversity and was calculated using the Vegan package (version 2.6-4) (32). The coordinates for the NMDS plot were computed using the metaMDS function from the Vegan package. All figures were generated using OriginPro (version 2024-SR1) (OriginLab Corporation, Northampton, MA, USA).

## RESULTS

### Sequencing performance for bacterial isolates

Overall, we processed 144 bacterial isolates using our ONT-based workflow with the aim to compare its performance for species-level annotation to the current gold-standard technique, MALDI-TOF MS. As shown in Table S7, after processing the raw sequencing reads, we obtained average Phred scores of 14+ for simplex (>74% above Q20) and duplex (>85% above Q20) data. For all simplex workflow options (S1BC and S2BC), over 95% of samples passed the tentative threshold of 10 reads. Duplex workflows achieved an average of 93% passed samples (D1BC) and 89% passed samples (D2BC), respectively. On average, the 1BC workflows resulted in more reads per sample, compared to 2BC (S1BC: 2,446 and D1BC: 183; S2BC: 2,004 and D2BC: 129). Consequently, 1BC reached higher efficiency values compared to 2BC (S1BC: 49% and D1BC: 41%; S2BC: 41% and D2BC: 21%). PCR efficiency for each sample pool (sharing the same native ONT barcode) was calculated as the sum of reads containing both a single/double PCR barcode and the native ONT barcode, divided by the total reads for the corresponding native ONT barcode.

### Cross-validation of S2BC data via MALDI-TOF MS

**Fig 3:**
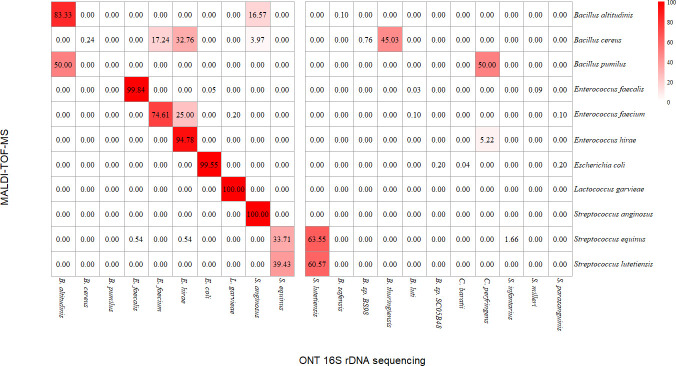
Heatmap visualizing the percentage of ONT 16S rDNA amplicon reads matching the MALDI-TOF MS identification result for each tested species, assuming MALDI-TOF MS as a gold standard.

As our workflow yields more simplex data, which is crucial considering scalability and multiplexing capabilities, we explored the potential of S2BC reads for bacterial identification. For 47/144 samples, we therefore compared the identification results of our nanopore-based workflow with identification using a MALDI-TOF MS platform. We set a purity cutoff of 90% for each sample— meaning at least 90% of all S2BC reads identified via EMU had to be assigned to the same species. Of the 47 samples, 7 samples did not meet this requirement, as less than 90% of annotated reads corresponded to the same species. Identification results from both techniques matched for 36/40 samples. Hence, we obtained a positive predictive value of 0.90 using our nanopore-based workflow, and we found a strong correlation between both techniques (Cramér’s *V* = 0.857 and Theil’s *U* = 0.316). To account for the absence of true negatives in our data set—and therefore calculation of specificity—a 16S amplicon PCR using growth medium as a negative control was performed, which did not yield any PCR product. To visualize the concordance between the two techniques, we constructed a heatmap (Fig. 3) showing the average percentage of matching ONT sequencing reads for each tested species. For *Enterococcus faecalis, Enterococcus hirae, Escherichia coli, Lactococcus garvieae, and Streptococcus anginosus,* the average of matching reads lies above 90%.

### Identification of bacterial isolates and phylogenetic tree-building using D1BC data

**Fig 4:**
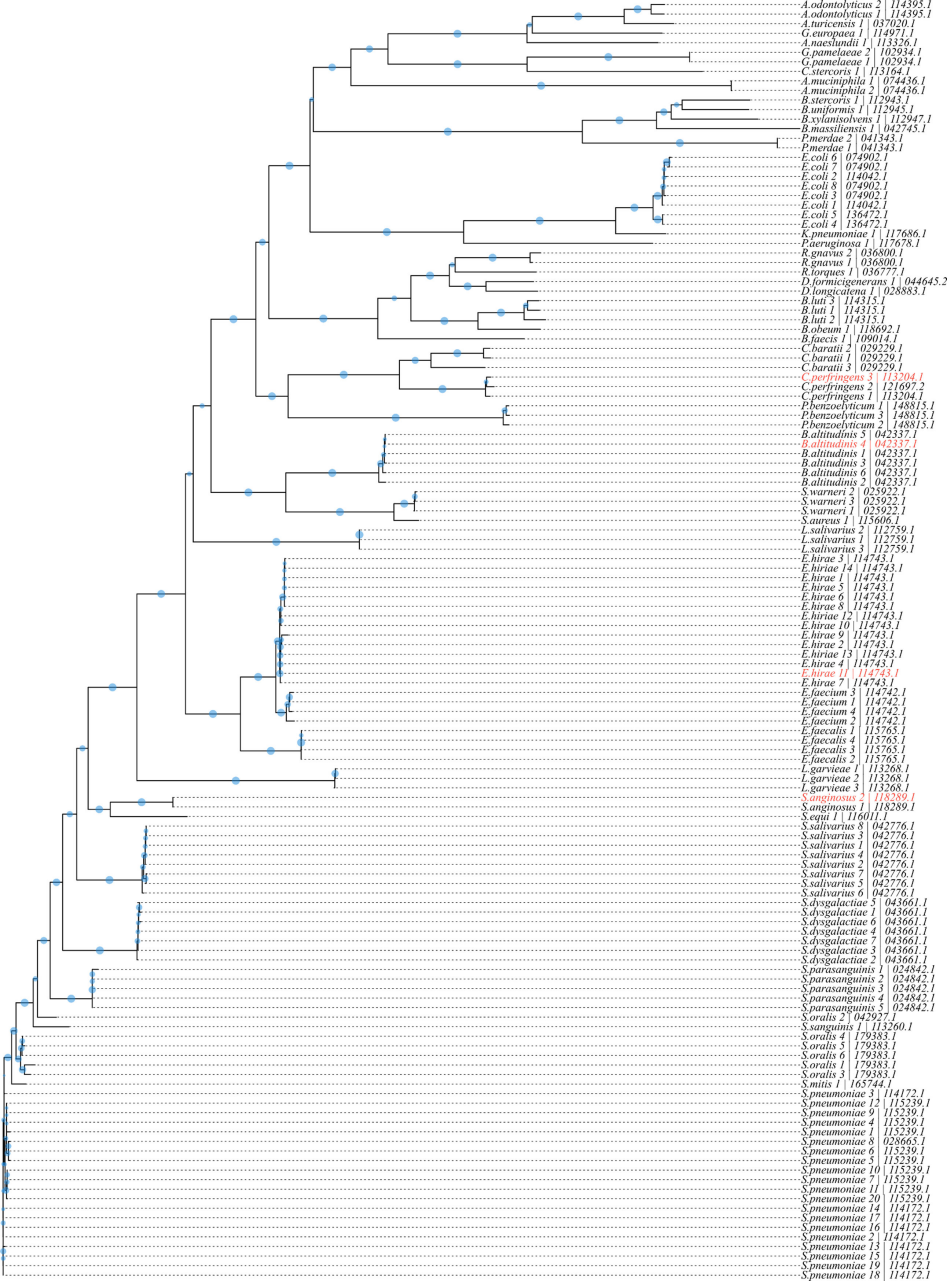
Phylogenetic tree constructed via the multiple sequence alignment of 134 full-length 16S rDNA consensus sequences in ClustalW2. Branch length is based on the distance matrix generated from pairwise alignment scores. Bootstrapping values (*n* = 1,000) are indicated as blue circles on the branches (larger circles correspond to larger bootstrapping values). Isolates marked in red were wrongly identified by our sequencing workflow.

In total, 134/144 bacterial isolates (7/144 excluded from MALDI analysis due to contamination and therefore excluded here, 3/144 did not result in any D1BC reads) spanning across 41 species, including closely related and more distant species, were sequenced to assess the taxonomic range of our assay and assess whether D1BC reads were of sufficient quality and quantity for species-level phylogenetic tree building. We obtained separated clusters on both genus and species level, as shown in Fig. 4. Furthermore, Emu annotations and blast annotations of the medaka consensus sequences matched in 122/134 cases. Mismatches comprised *E. coli* 3, 6–8 (Emu: *E. coli*, blast: *Escherichia fergusonii*), *E. coli* 4–5 (Emu: *E. coli*, blast: *Escherichia marmotae*), *Streptococcus oralis* 1 (Emu: *S. oralis*, blast: *Streptococcus vulneris*), *S. mitis* 1 (Emu: *S. mitis*, blast: *Streptococcus gwangjuense*), and *S. oralis* 3–6 (Emu: *S. oralis*, blast: *Streptococcus vulneris*).

### Performance of full-length 16S sequencing, using two published primer sets, to investigate different features of complex bacterial communities

**Fig 5:**
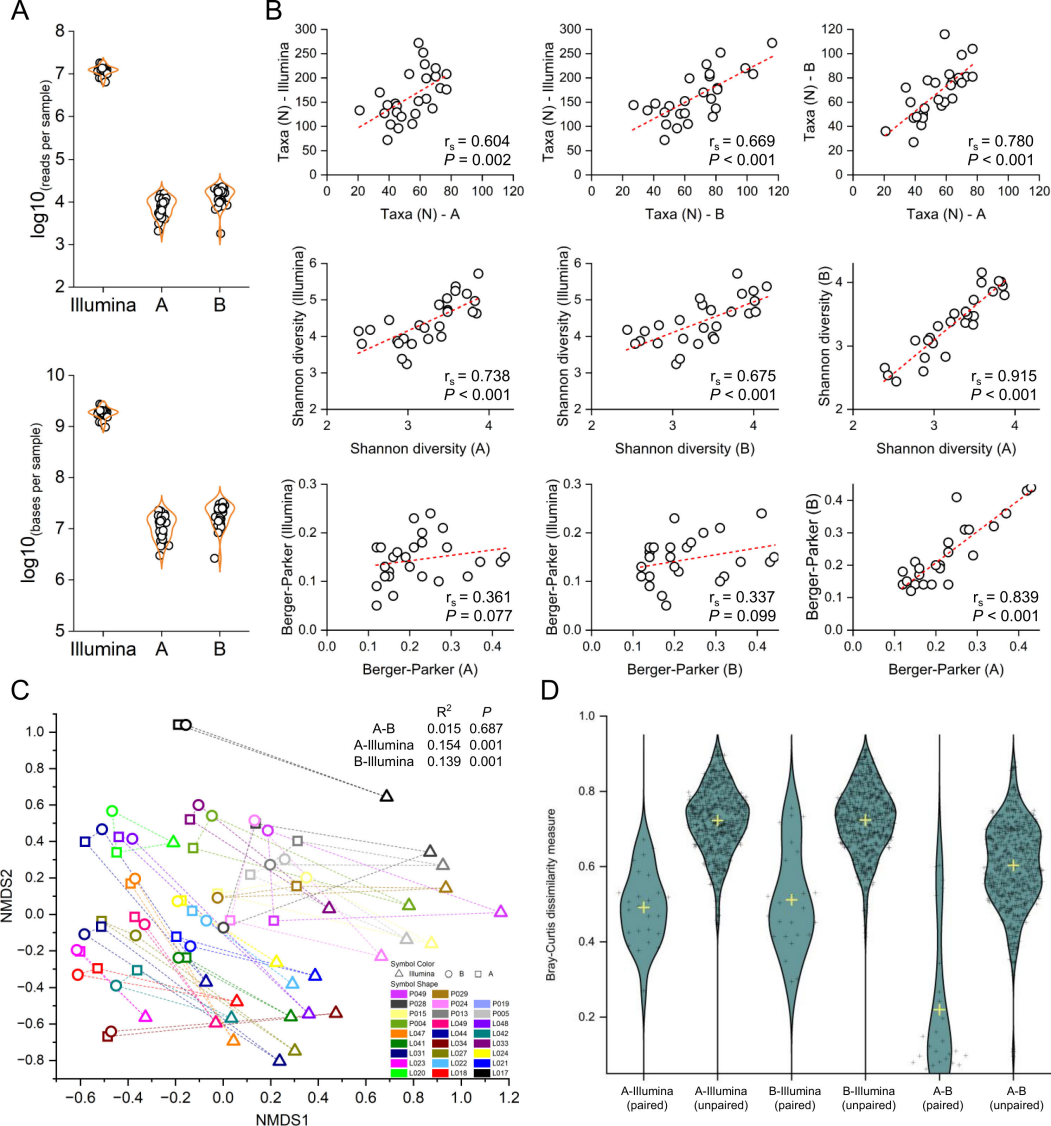
(A) Sequencing output per technology in sequenced reads and sequenced base pairs, per sample. (B) Spearman correlation of alpha diversity metrics between each sequencing technology. (C) Non-metric multidimensional scaling ordination plot based on Bray-Curtis dissimilarity and results of PERMANOVA analysis. (D) Bray-Curtis dissimilarity values between paired and unpaired samples and between sequencing technologies.

Using different full-length 16S primer pairs—named A (16) and B (19), we compared the performance of our Nanopore pipeline to investigate species-level microbial community metrics to a standard species-level profiling pipeline based on Illumina sequencing and the widely cited Metaphlan (29) taxonomic profiler. The libraries from 27 stool samples were sequenced in triplicate to mitigate sequencing bias and depth variations arising from flow-cell-specific differences in total sequencing output. An overview of the sequencing run metrics is given in Table S8. The averaged Phred scores for community sequencing were 17.0, 17.4, and 15.6 for A primers and 16.6, 17.2, and 17.4 for B primers, respectively (simplex basecalling). Two samples were discarded: one because Illumina library preparation failed, and the second because the total read count for 16S sequencing was below 1,000. Sequencing depth ranged between 6.4 M and 18.2 M paired-end reads per sample for Illumina, 2,081–15,920 reads for the A primers, and 1,816–22,717 reads for the B primers (Fig. 5A), resulting in 9.73E + 08–2.74E + 09 base pair (bp) per sample for Illumina, 3E + 06–2.31E + 07 bp for the A primers, and 2.64E + 06–3.28E + 07 bp for the B primers. The corresponding abundance tables are provided in Tables S9 and S10. We identified a significant correlation between the number of reads and the number of taxa for the A primer pair (*r*_*s*_ = 0.421, *P* = 0.037). However, no such correlation was observed for the other two alpha diversity metrics, namely Shannon diversity (SD) and Berger-Parker (BP) dominance. No correlation was found between sequencing depth and the various alpha diversity measures in the approach based on the B primer pair and Illumina sequencing. The effects of rarefaction for each 16S technique are shown in Fig. S1. When comparing species-level alpha diversity measures, we found a significant correlation between species richness for all comparison pairs, as shown in Fig. 5B. We also found strong correlation coefficients (*r*_*s*_ > 0.6, *P* < 0.001) for each comparison pair when comparing a composite metric of alpha diversity (SD). The dominance index (BP) did not correlate between Illumina and the two 16S primer pairs (*r*_*s*_ = 0.361 and 0.337; *P* = 0.077 and *P* = 0.099, respectively) but was strongly correlated between the two 16S-based approaches (*r*_*s*_ = 0.839, *P* < 0.001). For inter-samples and inter-techniques comparisons (= beta diversity), the first step was to merge the taxonomy for data sets analyzed with different sequencing techniques and taxonomic profilers (= Illumina versus 16S-based analyses). Since overall diversity was quite low for the ONT-based profiling, we aimed to merge the taxonomy of the 100 most abundant species observed with the A as well as B primers to the taxonomy obtained in the Illumina pipeline. The merging pipeline and the corresponding results are summarized in Fig. S2.

Using the Bray-Curtis dissimilarity measure, we performed a PERMANOVA analysis to compare community-wide composition (Fig. 5C) and found that the A-B pair was not significantly different (*R*^2^ = 0.015 and *P* = 0.687). We found significant differences at the community level between Illumina and pair A (*P* < 0.001) as well as pair B (*P* < 0.001) but low *R*^2^ values (0.154 and 0.139, respectively), thus indicating that the sequencing technique variable explains only a small proportion of the variability in the taxonomic profiles. Finally, we also compared the Bray-Curtis dissimilarity values of sample pairs across the sequencing techniques and found that pairwise dissimilarity is lower between sample pairs than between unpaired samples for all comparison groups (Fig. 5D). However, the pairwise dissimilarity is significantly lower between both 16S methods than between Illumina and both 16S sequencing.

**Fig 6:**
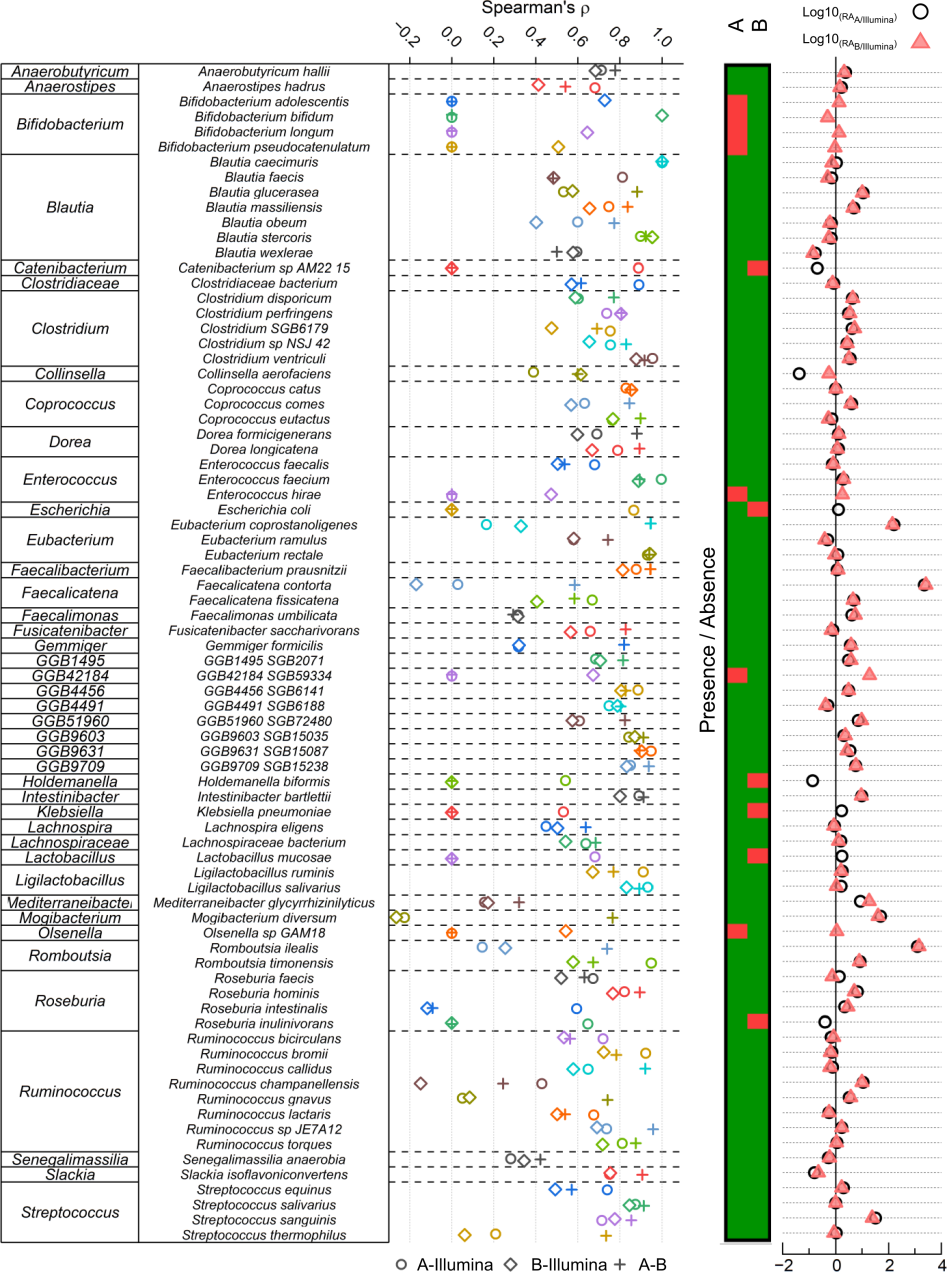
Species-level comparison between sequencing approaches. The left panel indicates the Spearman correlation values of each species between the different sequencing approaches. The middle panel indicates the presence/absence of each species for 16S-based ONT sequencing approaches. Red indicates that the species was not detected, while green indicates that it was. All species presented here were detected using Illumina sequencing. The right panel shows the normalized relative abundance fold change between Illumina and both 16S-based ONT approaches.

We then set to provide an in-depth comparison of species-level relative abundances between each analytical strategy. Using the tables with unified taxonomies, we compared the correlation coefficients of each species between the three sequencing protocols (Fig. 6, left panel). We observed species- and technique-specific variability in the Spearman correlation coefficient. Overall, the average Spearman correlation values were similar between Illumina-A and A-B [average (*r*_*s*_) = 0.600 and 0.619, respectively] and slightly lower in the Illumina-B comparison [average (*r*_*s*_) = 0.533], indicating a better agreement in terms of species relative abundances between the Illumina-based and the 16S/A-based profiling. Interestingly, however, there were some differences in terms of the presence/absence of different species between the two 16S-based profiling techniques (Fig. 6, middle panel). For instance, the A primer pair did not pick up any signal from the *Bifidobacterium* genus, while the B primers were able to detect four different *Bifidobacterium* species. Finally, we also showed the fold change for each species between each technique (Fig. 6, right panel).

## DISCUSSION

Our study presents a novel and flexible approach for sequencing full-length 16S rDNA amplicons using the ONT sequencing platform and the latest v14 kit chemistry and Flongle Flow Cells (R10.4.1). This adaptable workflow yielded promising results across eight sequencing runs, demonstrating the feasibility of the workflow in bacterial identification (two runs) and microbiome characterization (six runs) in small batches at contained costs (Table S11).

The major strength of our workflow is its flexibility: if desired, one could adapt the provided code for the use of Emu with other databases such as SILVA (33) or SPINGO (34). Depending on the research question at hand, multiplexing capability, sequencing quality, and read depth can be tuned specifically via the given base calling and barcoding options (S1BC, S2BC, D1BC, and D2BC). To each option, there are noteworthy consequences: simplex basecalling will greatly increase read depth, but at the cost of sequencing quality, which appears to be in agreement with previously published studies, around Q15 (>74% above Q20) (12, 35, 36). While we can expect high-quality reads from duplex basecalling (>85% above Q20), related workflows will result in less reads per sample. Multiplexing more samples via 2BC is possible at the cost of reduced read depth, which is greater in the 1BC setups. Adaptations, such as switching to SpotOn or PromethION Flow Cells, can enhance throughput in scenarios requiring higher read depth.

While our sequencing results generally align with bacterial species identification data obtained through MALDI-TOF MS, a few divergences were observed, such as the misidentification of *Bacillus altitudinis, Bacillus pumilus,* and *Enterococcus faecium*. Taxonomically distant misidentifications, such as *B. altitudinis* that was misidentified as *Streptococcus lutetiensis*, could be attributed to glycerol stock contamination introduced between MALDI-TOF MS measurements and the sequencing experiment. Interestingly, two misidentifications still shared the correct genus (MALDI: *B. pumilus,* ONT: *B. altitudinis*; MALDI: *E. faecium,* ONT: *E. hirae*). As our workflow was able to identify and distinguish multiple samples belonging to these genera or even species and we could not detect decreased sequencing quality for these samples (*Q*-score 15–16), we argue that the glycerol of these particular samples also could have been contaminated.

When comparing full-length 16S sequencing to Illumina sequencing for species-level characterization of microbial communities, we found that while within-sample metrics remained consistent (e.g., Shannon diversity and Berger-Parker index), Illumina-based sequencing detected significantly greater taxonomic richness compared to its 16S-based counterparts. Based on the associations between alpha diversity metrics and sequencing depth found in this data set, increased per-sample sequencing depth is likely to unveil more low-abundance species, thereby enhancing richness metrics, but at an extra cost linked to decreased multiplexing capacity. Given that many studies and microbiome-based diagnostics prioritize composite metrics (e.g., SD) (37–39), both 16S-based approaches tested in our workflow offer a suitable and cost-effective alternative for characterizing alpha diversity metrics. In terms of inter-sample diversity, and in agreement with previously published studies (40), we observed disparities in taxonomic profiling among the various sequencing technologies (41, 42) and bioinformatics pipelines (43, 44), but the magnitude of these differences remained low (15.4% and 13.9% variability for primers A and B, respectively). While the reasons for differences observed between 16S-based and shotgun-based profiling have been described elsewhere (40), a significant proportion of this variability can be explained by the inability to perfectly match the taxonomies derived from the different analytical pipelines used in this study. Indeed, using our matching criteria, we were only able to match ~75% of species among the 100 most abundant species observed with 16S-based approaches. Hence, the differences observed in this study are likely over-estimated and using analytical pipelines with unified taxonomies—which are not currently available for both data types—would result in further improved level of agreement between sequencing techniques. We also noted that the two 16S primer pairs yielded very similar results, in accordance with Matsuo et al*.* (19). It is noteworthy that although the overall composition differed between Illumina and the 16S-based methods, the relative abundances of the most prevalent taxa remained consistent. Therefore, if cost and portability are key factors, and low-abundance taxa are less relevant in specific applications, our proposed approach could be utilized for rapid and cost-effective species-level profiling of complex microbial communities.

At the individual species level, we found good correlation values between Illumina sequencing and the two 16S-based techniques. On average, the A primer showed a better correlation with Illumina in terms of species-level correlation than the B set. However, in terms of presence/absence, the B primer pair picked up a richer signal, closer to that of Illumina than the A set. For instance, we did not observe any *Bifidobacterium*-related signal in the A data set but found four *Bifidobacterium* species when using the more recent B primer set, agreeing with the results described by Matsuo et al. (19). Additionally, we observed species-specific disparities in several genera. An example of such is the *Faecalicatena* and *Romboutsia* genera, each represented by two species (*Faecalicatena contorta* and *Faecalicatena fissicatena*, and *Romboutsia ilealis* and *Romboutsia timonensis*). In each case, the normalized fold change is high [log10(FC) > 3] for one species but low [log10(FC) < 1] for the other species. Hence, while both A- and B-based approaches are overall comparable in terms of species-level detection, it is essential to carefully weigh these factors and consider their suitability for specific applications.

Our study has several important limitations. First, the selection of bacterial isolates sequenced throughout the study appears to be taxonomically limited and overshadowed by *Enterococcus* and *Streptococcus* species. Therefore, effective detection of untested species might not be guaranteed. However, the agreement of species-level annotations with MALDI-TOF MS data and the robust performance of our workflow in characterization of more complex bacterial communities demonstrated that a vast number of different taxa is in fact detected and correctly annotated. Second, we acknowledge that there are differences in the sequencing quality of simplex data basecalled by Dorado version 0.2.4 (bacterial isolate data; Phred score ~15) versus Dorado version 0.5.0 (stool sample data; Phred score ~17). Despite this difference, simplex data were of adequate quality to annotate bacterial isolate reads with high confidence. Finally, while the use of different reference databases and marker gene sets presents limitations, we attempted to systematically merge the taxonomies to facilitate a more coherent comparison. A species-level annotation was prioritized because species-level microbiome characterization provides several key advantages: it offers greater resolution, allows for more precise functional predictions, enables the identification of specific microbial interactions, and is crucial for studying disease-microbiome associations. This level of detail is essential for understanding the role of individual microbial species in health and disease, which can lead to more targeted therapeutic interventions and a better understanding of microbial contributions to disease mechanisms.

To conclude, our workflow allows for flexible full-length 16S sequencing using the latest v14 ONT chemistry. For large-scale approaches, there are more cost-effective short-read sequencing platforms, such as the NovaSeq X. However, our long-read workflow allows for contained costs in smaller batches, due to sample pooling and usage of Flongle Flow Cells, and possesses additional advantages, such as its flexibility and portability. We demonstrated its robust performance regarding annotation of bacterial isolates (compared to MALDI-TOF MS) and complex bacterial communities (compared to Illumina shotgun sequencing). The provided Nextflow pipeline simplifies data analysis and is modifiable and expandable, while by applying different PCR primers and databases, our protocol can be tailored to diverse research needs. We conclude that S1BC is a suitable option for applications where maximal read depth is needed (e.g., microbiome characterization) and S2BC and D1BC are targeted at large-scale and multiplexed bacterial identification scenarios—leaving the choice between (i) more read depth and multiplexing (S2BC) or (ii) higher quality reads (D1BC). While modifications are essential for high-throughput applications, the core strengths of our approach lie in its adaptability, flexibility, and potential for expansion—hopefully making it a versatile tool for the research community.

## Data Availability

The Nextflow pipeline is available under https://github.com/STPHxBioinformatics/ndp. The raw ONT sequencing data (16S rRNA amplicon sequencing) generated in this study have been deposited in the NCBI Short Read Archive under the accession PRJNA1082268.

## SUPPLEMENTAL MATERIAL

Supplemental Material msystems.00859-24-s0001.pdf Supplemental tables and figures.

ASM does not own the copyrights to Supplemental Material that may be linked to, or accessed through, an article. The authors have granted ASM a non-exclusive, world-wide license to publish the Supplemental Material files. Please contact the corresponding author directly for reuse.
