# Supplementary material for: A novel barcoded nanopore sequencing workflow of high-quality, full-length bacterial 16S amplicons for taxonomic annotation of bacterial isolates and complex microbial communities
Source: mSystems. 2024 Sep 10;9(10):e00859-24. doi: 10.1128/msystems.00859-24 (PMC11494973; doi:10.1128/msystems.00859-24)
Supplement: Supplemental Material — Supplemental tables and figures. [file msystems.00859-24-s0001.pdf]

Supplementary Figures and Tables

Supplementary Table 1: Bacterial isolates used in the study (n = 144).

Identity	Number of isolates	Origin
Actinomyces naeslundii	1	Institute for Infectious Diseases, Berne, Switzerland
Actinomyces odontolyticus	2	Institute for Infectious Diseases, Berne, Switzerland
Actinomyces turicensis	1	Institute for Infectious Diseases, Berne, Switzerland
Akkermansia muciniphila	3	Ordered (https://www.dsmz.de; DSM 22959)
Bacillus altitudinis	6	Isolated from stool at SwissTPH, Allschwil, Switzerland
sp. of Bacillus cereus group	2	Isolated from stool at SwissTPH, Allschwil, Switzerland
Bacillus pumilus	2	Isolated from stool at SwissTPH, Allschwil, Switzerland
Bacteroides massiliensis	1	Ordered (https://www.dsmz.de; DSM 17679)
Bacteroides stercoris	1	Ordered (https://www.dsmz.de; DSM 19555)
Bacteroides uniformis	1	Ordered (https://www.dsmz.de; DSM 6597)
Bacteroides xylanisolvens	1	Ordered (https://www.dsmz.de; DSM 18836)
Blautia faecis	1	Isolated from stool at University of Lausanne, Lausanne, Switzerland
Blautia luti	3	Isolated from stool at University of Lausanne, Lausanne, Switzerland
Blautia obeum	1	Isolated from stool at University of Lausanne, Lausanne, Switzerland
Clostridium baratii	3	Isolated from stool at University of Lausanne, Lausanne, Switzerland (n = 2) Isolated as contaminant at SwissTPH, Allschwil, Switzerland (n = 1)
Clostridium perfringens	2	Isolated from stool at University of Lausanne, Lausanne, Switzerland
Collinsella stercoris	1	Ordered (https://www.dsmz.de; DSM 13279)
Dorea formicigenerans	2	Obtained from University of Lausanne, Lausanne, Switzerland (JCM 31256; n = 1) Ordered (https://www.dsmz.de; DSM 3992, n = 1)
Dorea longicatena	1	Ordered (https://www.dsmz.de; DSM 13814)
Enterococcus faecalis	4	Isolated from stool at SwissTPH, Allschwil, Switzerland
Enterococcus faecium	5	Isolated from stool at SwissTPH, Allschwil, Switzerland
Enterococcus hirae	15	Isolated from stool at SwissTPH, Allschwil, Switzerland
Escherichia coli	9	Isolated from stool at SwissTPH, Allschwil, Switzerland (n = 5) Ordered (https://www.dsmz.de; DSM 30083, n = 1) Isolated from stool at University of Lausanne, Lausanne, Switzerland (n = 3)
Gleimia europaeus	1	Institute for Infectious Diseases, Berne, Switzerland
Gordonibacter pamelaeae	2	Ordered (https://www.dsmz.de; DSM 19378)
sp. of Klebsiella pneumoniae group	1	Ordered (https://www.dsmz.de; DSM 30104)
Lactobacillus salivarius	3	Ordered (https://www.dsmz.de; DSM 20555)
Lactococcus garvieae	3	Isolated from stool at SwissTPH, Allschwil, Switzerland
Parabacteroides merdae	2	Ordered (https://www.dsmz.de; DSM 19495)
Paraclostridium benzoelyticum	3	Isolated from stool at SwissTPH, Allschwil, Switzerland
Pseudomonas aeruginosa	1	Ordered (https://www.dsmz.de; DSM 1128)
Ruminococcus gnavus	2	Ordered (https://www.dsmz.de; DSM 108212)
Ruminococcus torques	1	Isolated from stool at SwissTPH, Allschwil, Switzerland

sp. of Staphylococcus aureus group	1	Ordered (https://www.dsmz.de; DSM 20231)
Staphylococcus warneri	3	Isolated from stool at SwissTPH, Allschwil, Switzerland
Streptococcus anginosus	1	Isolated from stool at SwissTPH, Allschwil, Switzerland
Streptococcus dysgalactiae	7	Institute for Infectious Diseases, Berne, Switzerland
Streptococcus equi	1	Institute for Infectious Diseases, Berne, Switzerland
Streptococcus equinus	2	Isolated from stool at SwissTPH, Allschwil, Switzerland
Streptococcus lutetiensis	1	Isolated from stool at SwissTPH, Allschwil, Switzerland
Streptococcus mitis	1	Institute for Infectious Diseases, Berne, Switzerland
Streptococcus oralis	6	Institute for Infectious Diseases, Berne, Switzerland
Streptococcus parasanguinis	5	Ordered (https://www.dsmz.de; DSM 6778)
Streptococcus pneumoniae	20	Institute for Infectious Diseases, Berne, Switzerland
Streptococcus salivarius	8	Ordered (https://www.dsmz.de; DSM 20067)
Streptococcus sanguinis	1	Isolated from stool at University of Lausanne, Lausanne, Switzerland

Supplementary Table 2: List of primers (A) used for the amplification of bacterial 16S rDNA in bacterial isolate samples and stool samples that were adapted from Urban et al. (7). Indicated in bold are the 12 bp long barcodes used for demultiplexing, followed by either a 27F or 1492R binding sequence.

Primer	Primer Sequence
P01-FWD	**GAGCCCGTTCCG**AGAGTTTGATCMTGGCTCAG
P02-FWD	**TGGCACCGATTA**AGAGTTTGATCMTGGCTCAG
P03-FWD	**GACATACAATGA**AGAGTTTGATCMTGGCTCAG
P04-FWD	**ATGGTCTACTAC**AGAGTTTGATCMTGGCTCAG
P05-FWD	**CCACTTGGATAG**AGAGTTTGATCMTGGCTCAG
P06-FWD	**CGATTATGGCAC**AGAGTTTGATCMTGGCTCAG
P07-FWD	**CTTACGAGGCAT**AGAGTTTGATCMTGGCTCAG
P08-FWD	**GTCCACCCTGGG**AGAGTTTGATCMTGGCTCAG
P01-REV	**GAGCCCGTTCCG**CGGTTACCTTGTTACGACTT
P02-REV	**TGGCACCGATTA**CGGTTACCTTGTTACGACTT
P03-REV	**GACATACAATGA**CGGTTACCTTGTTACGACTT
P04-REV	**ATGGTCTACTAC**CGGTTACCTTGTTACGACTT
P05-REV	**CCACTTGGATAG**CGGTTACCTTGTTACGACTT
P06-REV	**CGATTATGGCAC**CGGTTACCTTGTTACGACTT
P07-REV	**CTTACGAGGCAT**CGGTTACCTTGTTACGACTT
P08-REV	**GTCCACCCTGGG**CGGTTACCTTGTTACGACTT

Supplementary Table 3: List of primers (B) used for the amplification of bacterial 16S rDNA in stool samples that were adapted from Matsuo et al. (10) and were used for microbiome characterization. Indicated in bold are the 12 bp long barcodes used for demultiplexing, followed by either a 27F or 1492R binding sequence containing multiple degenerate bases (R, Y, M).

**Primer**	**Primer Sequence**
P01-FWD	**GAGCCCGTTCCG**AGRGTTYGATYMTGGCTCAG
P02-FWD	**TGGCACCGATTA**AGRGTTYGATYMTGGCTCAG
P03-FWD	**GACATACAATGA**AGRGTTYGATYMTGGCTCAG
P04-FWD	**ATGGTCTACTAC**AGRGTTYGATYMTGGCTCAG
P05-FWD	**CCACTTGGATAG**AGRGTTYGATYMTGGCTCAG
P06-FWD	**CGATTATGGCAC**AGRGTTYGATYMTGGCTCAG
P07-FWD	**CTTACGAGGCAT**AGRGTTYGATYMTGGCTCAG
P08-FWD	**GTCCACCCTGGG**AGRGTTYGATYMTGGCTCAG
P01-REV	**GAGCCCGTTCCG**CGGYTACCTTGTTACGACTT
P02-REV	**TGGCACCGATTA**CGGYTACCTTGTTACGACTT
P03-REV	**GACATACAATGA**CGGYTACCTTGTTACGACTT
P04-REV	**ATGGTCTACTAC**CGGYTACCTTGTTACGACTT
P05-REV	**CCACTTGGATAG**CGGYTACCTTGTTACGACTT
P06-REV	**CGATTATGGCAC**CGGYTACCTTGTTACGACTT
P07-REV	**CTTACGAGGCAT**CGGYTACCTTGTTACGACTT
P08-REV	**GTCCACCCTGGG**CGGYTACCTTGTTACGACTT

Supplementary Table 4: Reagents and volumes used per well in the 16S rDNA PCR reaction. Primers and DNA template were diluted in nuclease-free water.

**Reagent**	**Volume per reaction [µl]**
Nuclease-free water	8.5
NEB LongAmp Hot Start *Taq* 2x Master Mix	12.5
16S forward primer (10µM)	1
16S reverse primer (10µM)	1
DNA template (1:10 diluted)	2
Reaction volume	25

Supplementary Table 5: PCR setup conditions used for the 16S amplicon barcoding reaction.

Cycling conditions		
**Temperature**	**Time**	
94°C	30s	
94°C	30s	30-35 x
65°C	15s	
65°C	50s	
65°C	10min	

Supplementary Table 6: Chosen settings for performed sequencing runs using a Flongle Cell on a MinION Mk1C. Runtime was entirely dependent on the Flongle Flow cell and number of total reads reaching a plateau phase.

**Runtime**	10 - 22h
**Minimum length**	200bp
**Minimum Q-score**	9
**Basecalling**	Fast Basecalling
**Barcoding**	On (default)
**File: Raw reads**	POD5
**File: Basecalled**	FastQ

Supplementary Table 7: Overview of the two performed bacterial isolate sequencing runs for the four basecalling and barcoding options (S = simplex; D = duplex; S1BC = simplex basecalled, matching PCR barcodes; S2BC = simplex basecalled, different PCR barcodes; D1BC = duplex basecalled, matching PCR barcodes; D2BC = duplex basecalled, different PCR barcodes). Samples with more than 10 reads were classified as "Passed samples".

**Run metrics**			**average**
Primer set	A	A	
Number of pooled samples (n)	75	75	
Number of initial flongle pores	78	75	
Number of sequenced nucleotides [Mb]	1270	634	952
Number of S / D reads (before filtering)	537464 / 67985	268509 / 39607	402987 / 53796
Number of S / D reads (after filtering)	482835 / 64492	248294 / 37834	365565 / 51163
Average S / D Phred score (after filtering)	15.5 / 16.2	14.9 / 15.8	
S / D read percentage > Q20 [%]	77 / 85	74 / 87	

**S1BC**			**average**
Average reads per sample	3289	1603	2446
Average barcoding efficiency [%]	50	48	49
Passed samples	74	73	73.5
Passed samples [%]	98.7	97.3	98.0

**S2BC**			**average**
Average reads per sample	2678	1329	2004
Average barcoding efficiency [%]	41	40	41
Passed samples	74	72	73
Passed samples [%]	98.7	96.0	97.3

**D1BC**			**average**
Average reads per sample	227	139	183
Average barcoding efficiency [%]	28	30	29
Passed samples	71	68	69.5
Passed samples [%]	94.7	90.7	92.7

**D2BC**			**average**
Average reads per sample	158	100	129
Average barcoding efficiency [%]	20	22	21
Passed samples	69	64	66.5
Passed samples [%]	92.0	85.3	88.7

Supplementary Table 8: Overview of the six performed stool sample sequencing runs using the S1BC basecalling and barcoding option (A = full-length 16S primers adapted from Urban et al. (7); B = full length 16S primers adapted from Matsuo et al. (10); S1BC = simplex basecalled, matching PCR barcodes).

Run metrics							average (A)	average (B)
Primer set	A	A	A	B	B	B		
Number of pooled samples (n)	27	27	27	27	27	27		
Number of initial flongle pores	76	73	69	66	89	103	73	86
Number of simplex reads (before filtering)	200819	113003	265733	198244	307634	296618	193185	267499
Number of simplex reads (after filtering)	159607	103400	164508	152833	236713	195376	142505	194974
Average simplex Phred score (after filtering)	17.0	17.4	15.6	16.6	17.2	17.4		
Read percentage > Q20 [%]	84	85	78	82	84	85		
Average S1BC reads per sample	3429	2145	3229	3604	5674	4623	2934	4634
Average S1BC barcoding efficiency [%]	58%	56%	53%	65%	66%	65%	56%	65%

Supplementary Table 11: Estimated per sample cost for library preparation and sequencing of 24 (e.g. for microbiome characterization) or 80 (e.g. for single isolate annotation) samples using pooled approaches or unpooled approaches.

Estimated sequencing costs by approach			per sample costs	
**Reagent**	**kit costs**	**kit reactions**	**unpooled (n)**	**pooled (n / 8)**
Native Barcoding Kit 96 v14	$ 799.00	288	$ 2.77	$ 0.35
NEBNext® dA-Tailing Module (E6053L)	$ 650.00	384	$ 1.69	$ 0.21
Blunt/TA Ligase Master Mix (M0367L)	$ 600.00	125	$ 4.80	$ 0.60
NEBNext® Quick Ligation Module (E6056L)	$ 1’950.00	8000	$ 0.24	$ 0.24
cost per reaction			$ 9.51	$ 1.40
cost flongle flow cell			$ 65.00	$ 65.00
reaction cost for n = 80 samples			$ 825.86	$ 177.17
per sample cost for n = 80 samples			**$ 10.32**	**$ 2.21**
reaction cost for n = 24 samples			$ 293.26	$ 98.65
per sample cost for n = 24 samples			**$ 12.22**	**$ 4.11**

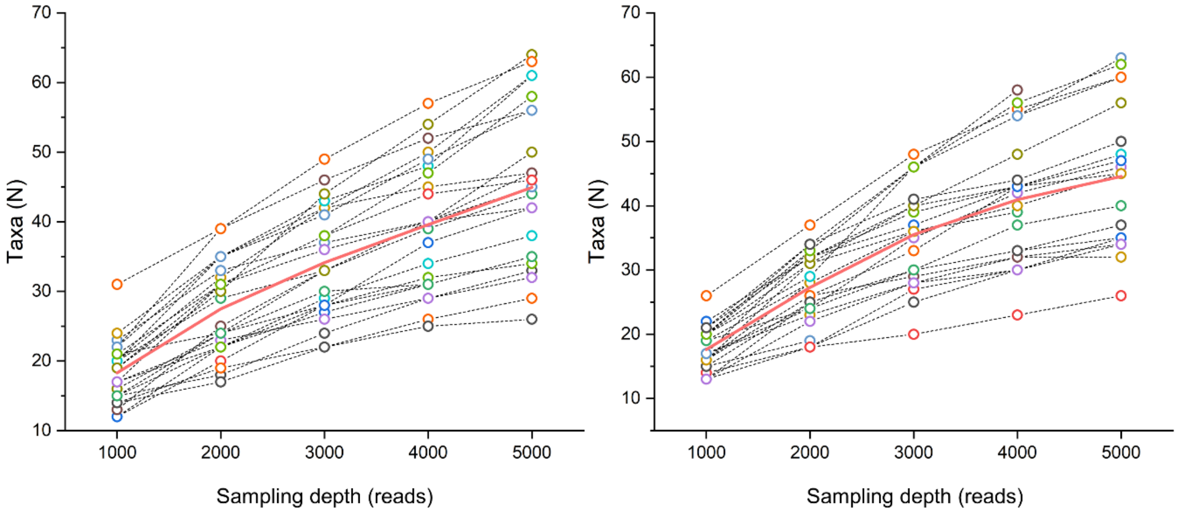

Supplementary Figure 1: Rarefaction curves of the 16S-based approaches, using A (left panel) and B (right panel) primer pairs. Each color represents one sample and its corresponding rarefactions. The average result of the rarefaction is depicted as a solid red line.

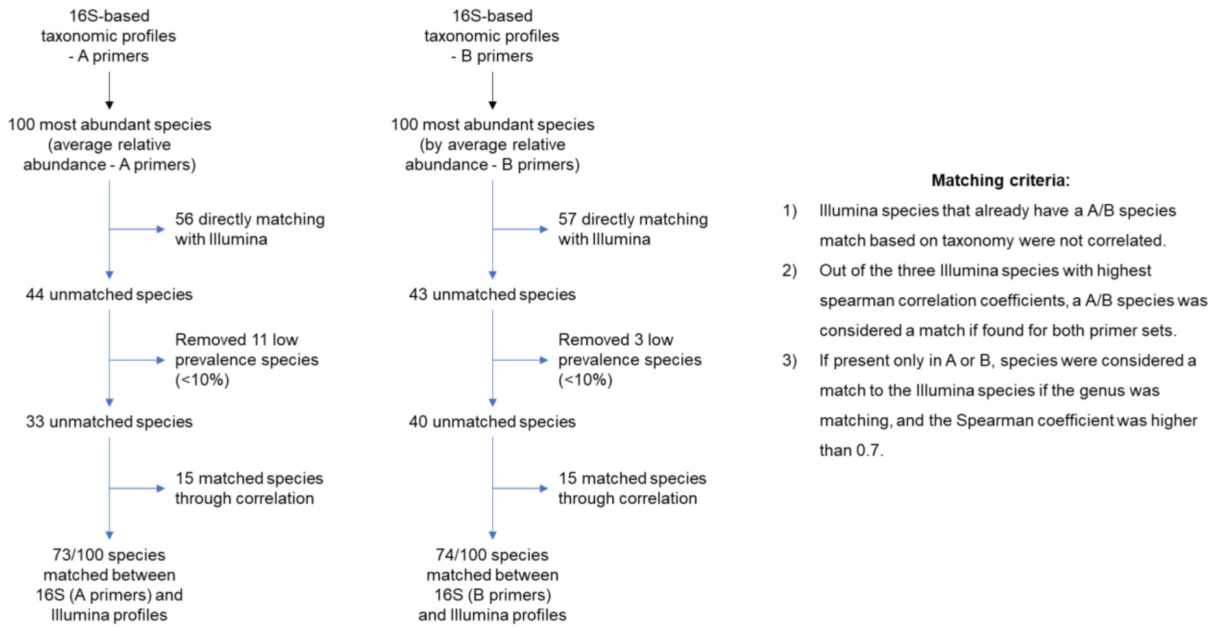

Supplementary Figure 2: Proportion of matched species between 16S-based and Illumina-based results and criteria used to merge divergent taxonomies resulting from different taxonomic profilers.
